# A Review of *FoxO1*-Regulated Metabolic Diseases and Related Drug Discoveries

**DOI:** 10.3390/cells9010184

**Published:** 2020-01-10

**Authors:** Shiming Peng, Wei Li, Nannan Hou, Niu Huang

**Affiliations:** 1 National Institute of Biological Sciences, Beijing 102206, China; Shiming.Peng@ucsf.edu (S.P.); hounannan@nibs.ac.cn (N.H.); 2 RPXDs (Suzhou) Co., Ltd., Suzhou 215123, China; liwei@rpxds.com; 3 Tsinghua Institute of Multidisciplinary Biomedical Research, Tsinghua University, Beijing 102206, China

**Keywords:** *FoxO1*, metabolic disease, drug discovery

## Abstract

*FoxO1* is a conserved transcription factor involved in energy metabolism. It is tightly regulated by modifications on its mRNA and protein and responds to environmental nutrient signals. *FoxO1* controls the transcription of downstream genes mediating metabolic regulation. Dysfunction of *FoxO1* pathways results in several metabolic diseases, including diabetes, obesity, non-alcoholic fatty liver disease, and atherosclerosis. Here, we summarize the mechanism of *FoxO1* regulation behind these diseases and *FoxO1*-related drug discoveries.

## 1. Introduction of *FoxO1*

**Figure 1:**
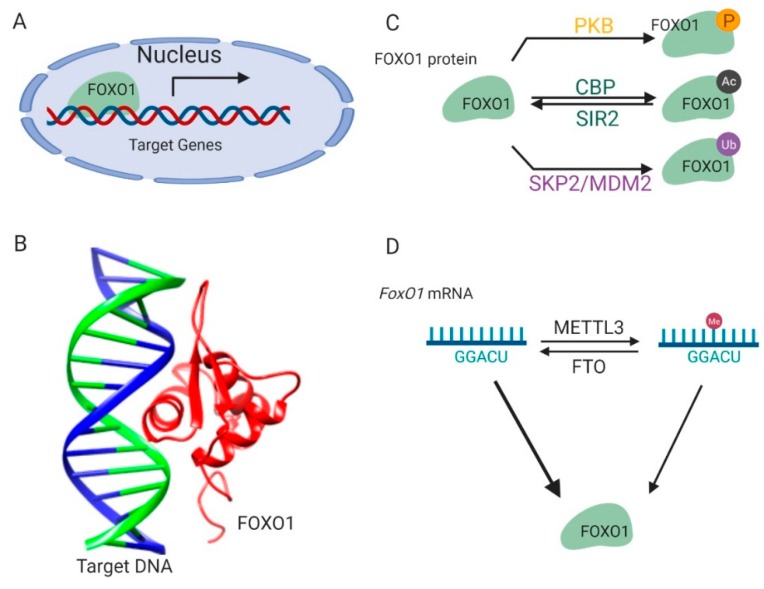
Regulation of *FOXO1* transcription activity. (**A**) FOXO1 binds to the promoters of target genes and stimulates their transcription in nucleus. (**B**) The complex structure of the FOXO1 protein and its target-binding DNA (Protein Data Bank id: 3CO6). (**C**) The post-translational modifications of the FOXO1 protein. (**D**) Methylations on the *FoxO1* mRNA regulate its expression. This figure was created with UCSF Chimera [29] and BioRender.com.

*Forkhead Box O* (*FoxO*) genes belong to a transcription factor (TF) family characterized by the presence of a conserved DNA-binding domain (the Forkhead box) in the N-terminal end of the protein [1,2,3] (Figure 1A). FOXOs bind the consensus DNA-binding element within the promoter of their target genes and regulate their transcription in response to external signal stimulation [4,5,6] (Figure 1B). There are four *FoxO* genes identified in mammals, including *FoxO1*, *FoxO3a*, *FoxO4,* and *FoxO6* [7]. In the 1990s, the *FoxO1* gene was first identified in studies of chromosomal translocations found in human tumors, including rhabdomyosarcomas and acute myeloid leukemias [1,3,8,9]. In addition to its important regulatory roles in oncogenesis [10], *FoxO1* also transcriptionally mediates pathways behind many metabolic diseases, including gluconeogenesis, glycogenolysis, adipogenesis, thermogenesis, and feeding behavior [11,12,13,14,15], which is the focus of this review article. 

## 2. Regulation of *FoxO1*

### 2.1. Protein Modifications

FOXO1 protein is sophisticatedly and strictly regulated by modifications on its protein and mRNA, which ensures that transcription of its downstream target genes is tightly responsive to environmental signals [16] (Figure 1C). Kinase-mediated phosphorylation of the FOXO1 protein in response to insulin or growth factors is a form of regulation [17] (Figure 1C). Phosphoinositide 3-kinase/protein kinase B (PI3K/PKB) phosphorylates the FOXO1 protein at three conserved residues, including Threonine 24, Serine 256, and Serine 319. These modifications result in disrupted interactions between the FOXO1 protein and its target DNA and lead to the translocation of the FOXO1 protein from the nucleus to the cytoplasm, thus suppressing FOXO1-dependent transcription. The FOXO1 protein can also be phosphorylated by c-Jun N-terminal kinase (JNK) or macrophage-stimulating 1 (Mst1) [18,19]. This phosphorylation results in the import of the FOXO1 protein from the cytoplasm to the nucleus, thereby antagonizing the action of PI3K/PKB. 

FOXO1 protein activity is also regulated by reversible acetylation modification on lysine residues (Figure 1C). They are acetylated by histone acetyltransferase cAMP-response element-binding protein (CREB)-binding protein (CBP) and deacetylated by NAD-dependent histone deacetylase silent information regulator 2 (Sir2) on conserved residues (Lys 242, Lys 245, and Lys 262) [20,21,22]. The positive charge of these lysine residues in FOXO1 contributes to DNA binding. Thus, acetylation at these residues attenuates the ability of FOXO1 to bind DNA and suppress transcription. Interestingly, it was also found that acetylation regulated the function of FOXO1 by influencing its sensitivity for phosphorylation.

The FOXO1 protein is polyubiquitinated by E3 ligases (Figure 1C) such as S-phase kinase-associated protein 2 (SKP2) and mouse double minute 2 homolog (MDM2), and targeted for protein degradation in human primary tumors and cancer cell lines [23,24,25]. The ubiquitination and proteasome degradation of the FOXO1 protein are essential in tumorigenesis and represent a viable target for cancer treatment. 

### 2.2. mRNA Methylation

In addition to these post-translational modifications on protein, *FoxO1* is also regulated by methylation modification on its mRNA [26,27,28] (Figure 1D). There are two specific adenosine sites in the coding sequence of the *FoxO1* mRNA [26,27]. The adenosine (A) at these sites can be methylated to N^6^-Methyladenosine (m^6^A) by methyltransferase like 3 (METTL3) protein, and m^6^A can be demethylated to A by fat mass and obesity-associated protein (FTO). These reversible m^6^A modifications participate in regulating *FoxO1* gene translation [28]. 

## 3. *FoxO1*-Regulating Mechanism Behind Diseases

### 3.1. Glucose Production in the Liver

**Figure 2:**
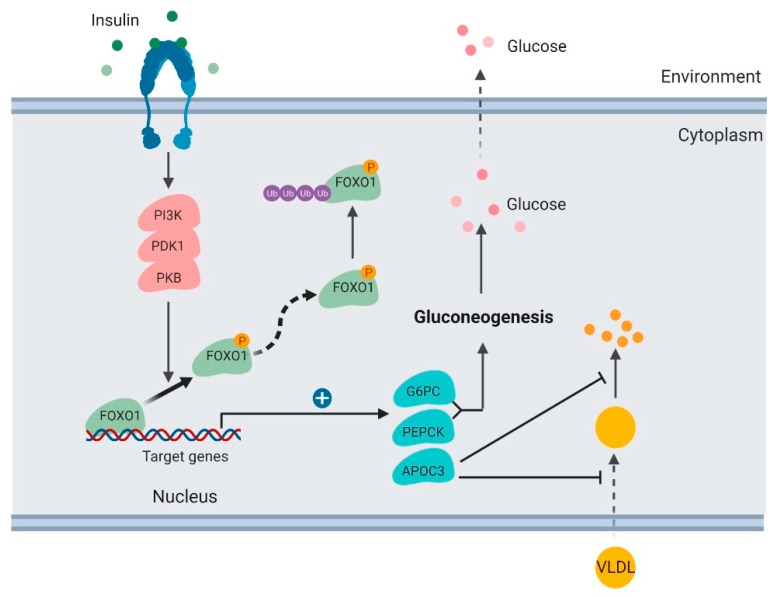
Regulation of glucose production and lipoprotein uptake by *FOXO1* in the liver (created with BioRender.com).

*FoxO1* regulates many important metabolic pathways in the liver, fat tissue, and hypothalamus [30,31]. It is well established that *FoxO1* regulates hepatic gluconeogenesis and glycogenolysis in response to an insulin signal in the blood (Figure 2). High concentration of insulin decreases blood glucose level by promoting glucose absorption after feeding and inhibits glucose production by hepatic gluconeogenesis and glycogenolysis in the fasting state. 

In the liver, insulin activates the PI3K/PKB signaling pathway and results in FOXO1 protein phosphorylation and degradation [32]. FOXO1 TF binds and promotes transcription of *glucose 6-phosphatase* (*G6PC*) and *phosphoenolpyruvate carboxykinase* (*PEPCK*), which are key enzymes stimulating gluconeogenesis and glycogenolysis [33,34]. When FOXO1 is suppressed, transcription of *G6PC* and *PEPCK* subsequently decreases, which consequently inhibits the rates of glucose production in the liver. 

It has been well established that *FOXO1* is a key mediator in the signaling pathway of insulin regulating hepatic gluconeogenesis. Hepatic *FOXO1* loss-of-function mutant suppresses *G6PC* and *PEPCK* expression, decreases hepatic gluconeogenesis, and improves fasting glycemia in diabetic *db/db* mice [32]. 

### 3.2. Lipoprotein Uptake in the Liver

*Apolipoprotein C-III* (*ApoC3*) is another downstream target of *FoxO1* that functions directly in plasma triglyceride metabolism [31,35]. *ApoC3* is secreted by the liver and enriched in very low-density lipoprotein (VLDL). It was reported to suppress hepatic uptake of VLDL and inhibit lipoprotein lipase [36]. *ApoC3* overexpression in humans functions in atherosclerosis [37].

FOXO1 binds to the ApoC3 promoter and enhances its transcription [35] (Figure 2). FOXO1 overexpression increases hepatic ApoC3 expression and elevates plasma triglyceride levels. FOXO1 loss-of-function mutation interferes with *ApoC3* expression in response to insulin stimulation. Insulin deficiency or resistance results in unrestrained ApoC3 expression and impaired triglyceride metabolism in the pathogenesis of atherosclerosis and hypertriglyceridemia.

### 3.3. Lipogenesis in the Liver

**Figure 3:**
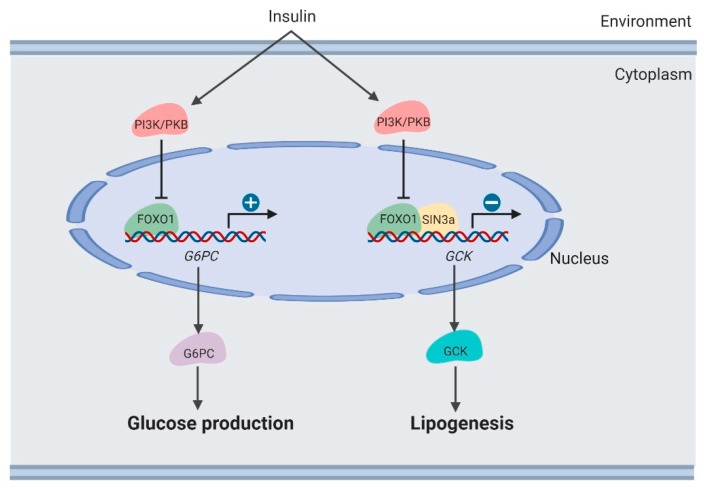
Regulation of lipogenesis by *FOXO1* in the liver (created with BioRender.com).

Clinically, it was observed that therapeutically decreasing blood glucose usually caused increased lipogenesis in the liver [11,38]. Lipogenesis is induced by suppressing the *FoxO1*-dependent inhibition of *glucokinase* (*Gck*) [39] (Figure 3). Hepatic *Gck* expression is associated with lipogenesis and fatty liver in humans. Increased *Gck* activity induced fatty liver and its metabolic consequences in humans.

Moreover, *SIN3 Transcription Regulator Family Member A* (*SIN3A*) was identified as an insulin-sensitive *FOXO1* corepressor of *Gck*. *SIN3A* knockout interferes with *Gck* expression regulated by *FOXO1* in response to environmental nutrients, while not affecting expression of other genes targeted by *FOXO1*, such as *G6PC*. These results may provide the opportunity to develop selective modulators of the *FoxO1* pathway, which removes adverse effects of hepatic lipogenesis in therapeutically treating diabetes [39].

### 3.4. Lipid Metabolism in Adipocytes

*FoxO1* binds to the promoter sites of *peroxisome proliferator-activated receptor gamma* (*PPARγ*) DNA and represses its transcription [40]. In response to insulin stimulation, FOXO1 protein is degraded and subsequently unable to prevent transcription of *PPARγ*. Because high levels of PPARγ protein initiate adipogenesis, *FoxO1* suppresses adipogenesis [41]. 

Moreover, the FOXO1 protein is a repressor of *uncoupling protein 1* (*Ucp1*) gene transcription [31,42]. UCP1 protein is a mitochondrial inner membrane proton channel linked to energy use that serves as a well-known biomarker of the thermogenesis state of adipose tissues [43,44,45]. *FoxO1* inhibition increases UCP1 expression, thereby augmenting thermogenesis and fat loss. Selectively inhibiting FTO by inhibitors decreases FOXO1 expression and reduces body weight and fat mass in a high-fat diet-induced obesity (DIO) mouse model. Suppressing FOXO1 expression increased the energy expenditure of mice. Thermogenesis in adipose tissue was induced by reduced FOXO1 expression, which was a cause of the decreased body weight and increased energy expenditure [28].

### 3.5. Feeding Behavior in the Hypothalamus

*FoxO1* in the central nervous system, mainly the hypothalamus, functions directly in integrating signals from peripheral tissues and mediating feeding behavior. Insulin and leptin are well-studied nutrient signals, integrating peripheral energy status to the hypothalamus. In the arcuate nucleus (ARC) of the hypothalamus, two neuronal populations express specific feeding-related neuropeptides, including pro-opiomelanocortin (POMC) and agouti-related peptide (AgRP) [46]. POMC suppresses appetite and decreases body weight. AgRP enhances food intake and increases body weight [47,48].

**Figure 4:**
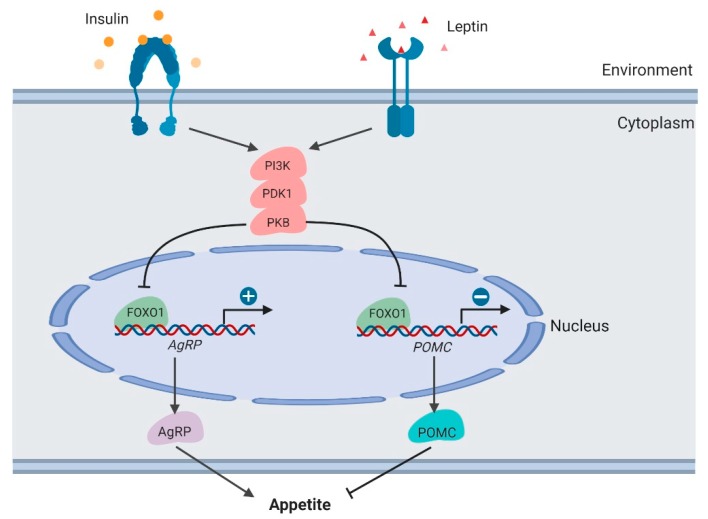
Regulation of appetite by *FOXO1* in the hypothalamus (created with BioRender.com).

The *PI3K/PDK1/PKB–FoxO1* signaling axis functions to integrate leptin and insulin signals to regulate POMC and AgRP secretion [49,50,51] (Figure 4). As the downstream target of these kinases, the FOXO1 protein is phosphorylated and inactivated in neurons, thus promoting POMC transcription and suppressing AgRP transcription. In mouse models, *PDK1* knockout in POMC neurons suppresses FOXO1 phosphorylation and degradation, thus suppressing POMC transcription. By contrast, in AgRP neurons, *PDK1* deletion results in enhanced AgRP expression and increased food intake. In the *PDK1*-knockout mouse models, *FoxO1* inactivation rescues these phenotypes, which demonstrates the crucial roles of *FoxO1* in regulating feeding behavior.

Furthermore, in a DIO rat model, the mechanism of *FoxO1* in regulating feeding behavior was confirmed [52]. In these rats, because of insulin resistance, the *PI3K/PDK1/PKB-FoxO1* regulatory axis was damaged, resulting in increasing the amount of FOXO1 protein and food intake. However, the intracerebroventricular (i.c.v.) micro-infusion of *FoxO1*-antisense oligonucleotides (*FoxO1*-ASOs) largely decreased the hypothalamic *FoxO1* expression, food intake, and body weight gain. These suggested that pharmacological inactivation of *FoxO1* may be used to suppress appetite and treat obesity.

## 4. *FoxO1*-Related Drug Discovery

Although some biologic therapies antagonizing FOXO1 activity were reported in treating cancers, wound repair, and cardiovascular diseases [53,54,55,56,57], many efforts are still focused on designing small-molecule compounds indicated for treating metabolic disorders [58].

### 4.1. FOXO1 Protein Inhibitors

**Figure 5:**
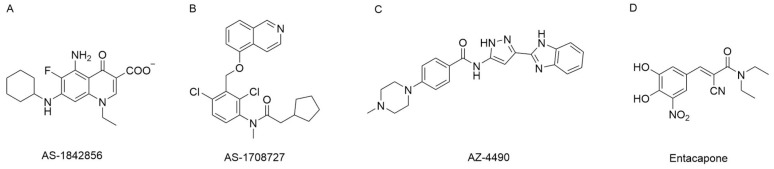
Inhibitors of the FOXO1 protein and *FOXO1* pathway. (**A**,**B**) Inhibitors of the FOXO1 protein. (**C**) Inhibitors of *FoxO1*-dependent glucose production without enhancing lipogenesis. (**D**) Entacapone suppressing FOXO1 protein expression through inhibiting fat mass and obesity-associated protein (FTO) enzymatic activity.

Selectively silencing FOXO1 has the potential to treat metabolic disorders. In a mass spectrometric affinity screening, a small-molecule compound, 5-amino-7-(cyclohexylamino)-1-ethyl-6-fluoro-4-oxo-1,4-dihydroquinoline-3-carboxylic acid (AS1842856), was identified to directly bind to the FOXO1 protein, and it selectively bound to the dephosphorylated activated form, but not the phosphorylated one (Figure 5A). Cellular assay showed that AS1842856 blocked the transcription activity of *FOXO1* with an IC_50_ of 0.03 μM but much less potently inhibited FOXO3a and FOXO4 with IC_50_ larger than 1 μM, which suggested that AS1842856 could be a potent and selective FOXO1 inhibitor [59,60]. In a diabetic *db/db* mouse model, oral administration of AS1842856 resulted in largely decreased plasma glucose after fasting and suppressed expression of gluconeogenic genes.

Focused on FOXO1 activity inhibition, one cell-based high-throughput screening of 170,000 small-molecule compounds resulted in a number of hits. Among them, FOXO1 inhibitor AS1708727 was identified with high oral activity, low clearance, and high liver distribution in mice [61] (Figure 5B). In a diabetic *db/db* mouse model, treatment with AS1708727 largely reduced blood glucose and blood triglyceride and decreased expression of hepatic *G6PC*, *PEPCK*, and *ApoC3*.

### 4.2. FoxO1 Pathway Modulators

Considering that FOXO1 is a TF, it is not easy to manipulate *FoxO1* through pharmacological approaches. Therefore, excepting the FOXO1 protein itself, researchers also pursue design modulators to selectively mediate the *FoxO1* pathway. After a small-molecule screening, a series of selective inhibitors of the *FoxO1* pathway were identified [39]. Interestingly, some compounds inhibited *FoxO1*-dependent *G6PC* transcription and enhanced *GCK* transcription in hepatocytes, while other compounds decreased *G6PC* transcription without significant influence on *GCK* transcription. These compounds inhibited glucose production without lipogenic activity. Among them, cpd-10 was reported by a group from AstraZeneca as AZ-4490, with an IC_50_ of 0.02 μM [62] (Figure 5C). 

**Figure 6:**
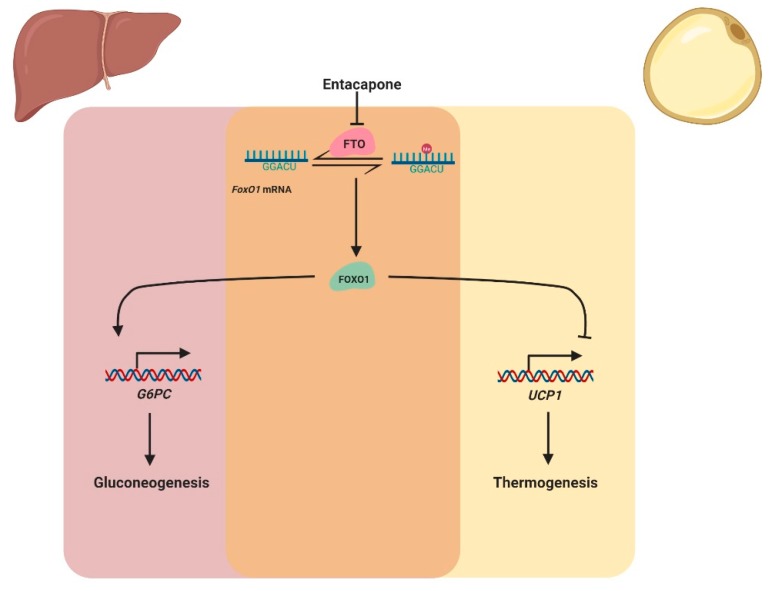
Entacapone suppresses gluconeogenesis in the liver and increases thermogenesis in adipocytes through reducing FOXO1 expression (created with BioRender.com).

In addition to experimental compound screening, selective inhibitors of the *FoxO1* pathway were designed rationally. FTO protein demethylases the m^6^A modifications on *FoxO1* mRNA and enhances its expression. Selective inhibition of FTO protein decreases the amount of FOXO1 protein in vivo. Via structure-based drug design, entacapone and its analogues were identified as selective small-molecule inhibitors of the FTO protein (Figure 5D). Entacapone decreased FOXO1 expression, thus suppressing gluconeogenesis and increasing thermogenesis in mouse models (Figure 6). Selectively suppressing *FoxO1* activity through the inhibition of FTO by entacapone provides the possibility to treat type II diabetes and obesity [28].
